# Immune context and treatment timing associated with seizure freedom after rituximab in autoimmune limbic encephalitis

**DOI:** 10.1016/j.isci.2026.116689

**Published:** 2026-07-10

**Authors:** Andre Dik, Nils Landmeyer, Noëmi Gmahl, Laura Bierhansl, Rakshit Dadarwal, Alina Kosfeld, Matthias Pawlowski, Tobias Brix, Catharina C. Gross, Gerd Meyer zu Hörste, Heinz Wiendl, Antje Bischof, Christian Elger, Sven G. Meuth, Stjepana Kovac

**Affiliations:** 1 Department of Neurology, University Hospital Münster, Münster, Germany; 2 Beta Klinik Bonn, Bonn, Germany; 3 Department of Neurology, University Hospital Freiburg, Freiburg, Germany

**Keywords:** autoimmune limbic encephalitis, seizure freedom, rituximab, immunotherapy timing, cerebrospinal fluid biomarkers, B-cell-mediated inflammation, autoimmune-associated epilepsy, treatment response predictors, antibody-associated encephalitis, clinical outcome measures

## Abstract

Seizure freedom represents a clinically meaningful outcome in autoimmune limbic encephalitis (ALE). Rituximab (RTX) is commonly used, but the factors associated with seizure outcomes remain unclear. We conducted a retrospective single-center cohort study including 107 adults with ALE and seizures treated between 2005 and 2024. Patients were stratified by RTX treatment, treatment timing, cerebrospinal fluid (CSF) findings, and seizure outcome. The primary endpoint was seizure freedom for ≥12 months. Overall, 72/107 patients (70.6%) achieved seizure freedom, including 18 RTX-treated patients. Earlier RTX initiation (< 12 months) was numerically associated with higher seizure freedom rates than later treatment (77.8% vs. 37.5%). In RTX-treated patients, positive CSF immune findings were associated with seizure freedom (100% vs. 62.5%). Combining early RTX initiation and positive CSF findings was associated with increased odds of seizure freedom. In multivariable analysis, RTX treatment was not independently associated with seizure freedom. These exploratory findings require prospective validation.

## Highlights

• Early rituximab initiation is associated with higher seizure freedom in ALE
• CSF immune features are associated with seizure outcomes following rituximab
• Treatment timing and immune context may improve stratification of seizure outcomes
• Seizure freedom represents a clinically meaningful outcome in ALE

## Graphical abstract

**Figure:**
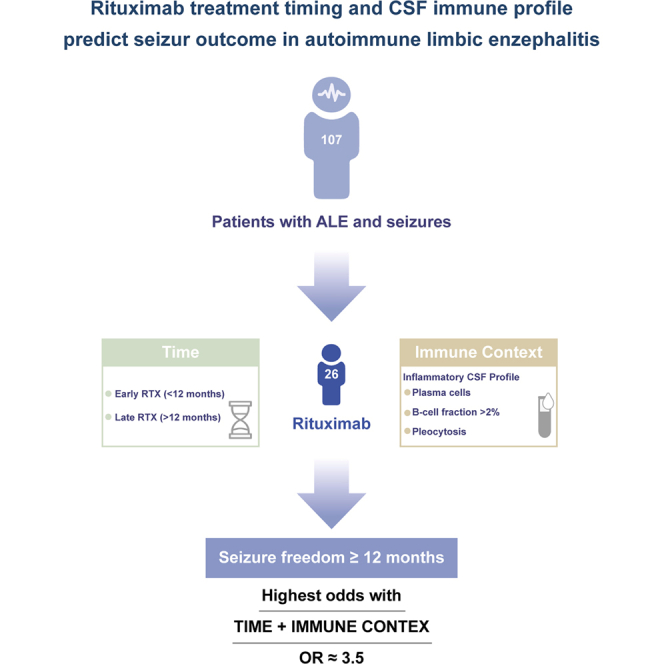

## Teaser

Clinical finding; Treatment; Immune system disorder

## Introduction

Autoimmune limbic encephalitis (ALE) often presents with seizures as a debilitating symptom of the disease. Seizure freedom after immune treatment often parallels resolution of inflammation and improvement of other symptoms such as cognitive decline and thus represents a quantifiable disease marker.^1^^,^^2^ Rituximab (RTX) has become an important escalation therapy in ALE, particularly in patients with ongoing disease activity despite first-line immunotherapy. Real-world data suggest favorable clinical outcomes in *N*-methyl-d-asoartate receptor (NMDAR) receptor encephalitis, yet its effectiveness in other antibody (AB)-positive or AB-negative ALE remains less well defined.^3^^,^^4^^,^^5^ While global disability scales such as the modified Rankin scale remain widely used in autoimmune encephalitis,^3^^,^^4^ they may insufficiently capture relevant non-motor and syndrome-specific manifestations. Disease-specific instruments such as the Clinical Assessment Scale in Autoimmune Encephalitis (CASE) provide a more granular assessment and include seizure activity as a clinically meaningful domain.^6^^,^^7^ In ALE, seizure freedom thus represents a valid and clinically meaningful disease marker, but reliable data on seizure freedom require detailed patient follow-up, which is often only available in tertiary epilepsy centers.^1^ Previous observations and clinical experience suggest that earlier RTX initiation and the presence of cerebrospinal fluid (CSF) findings reflecting an underlying immune activation state may be associated with more favorable seizure outcomes, rather than representing direct therapeutic targets.^3^^,^^5^ In addition, recent studies highlight the relevance of intrathecal immune phenotyping and B-cell-related activity in autoimmune encephalitis, suggesting that CSF immune profiles may provide context-specific information on disease activity and treatment responsiveness.^8^^,^^9^ However, systematic data addressing the impact of treatment timing on seizure outcomes in ALE are limited, and it remains unclear whether earlier intervention modifies long-term epileptogenic processes. Addressing these questions requires detailed longitudinal assessment of seizure activity, electroencephalography (EEG) findings, MRI evolution, CSF immune profiles, and treatment trajectories, information that is often unavailable in large administrative datasets and may be difficult to harmonize across multicenter cohorts in rare neuroimmunological disorders such as ALE.

It remains clinically relevant to assess the efficacy of RTX in ALE and to identify suitable candidates for this treatment, given that RTX affects the rate of infection-related hospitalization, which is higher compared to other immune treatments.^10^^,^^11^ We thus aimed to determine the impact of treatment timing and immunological context on seizure freedom after RTX therapy in a well-characterized cohort of patients with ALE. In contrast to prior studies primarily focusing on global functional outcomes, this study specifically evaluates seizure freedom as a clinically meaningful endpoint and integrates both temporal and biological factors. By combining treatment timing with immunological context, this study explores a potential stratified framework for RTX response in ALE and may help guide future individualized treatment strategies.

## Results

### Patient characteristics and cohort overview

**Figure 1:**
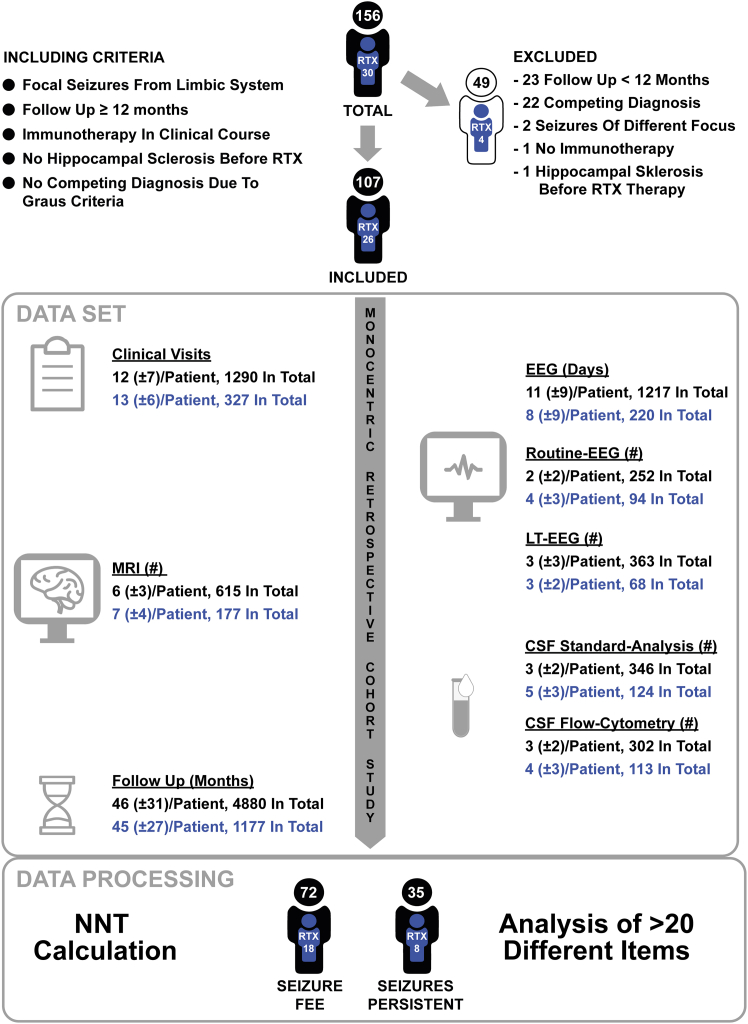
Study design Overview of patient inclusion, exclusion criteria, final cohort composition, and data processing workflow.

Of 107 patients with ALE (female 42%; median age 53 years) and seizures and a minimum follow-up of 12 months, 72 patients were seizure free, of whom 18 received RTX (25%). Seizure persistence was observed in 35 patients, 8 of whom received RTX (22.9%; Table S1). The study design, patient selection, and data processing workflow are summarized in Figure 1.

**Figure 2:**
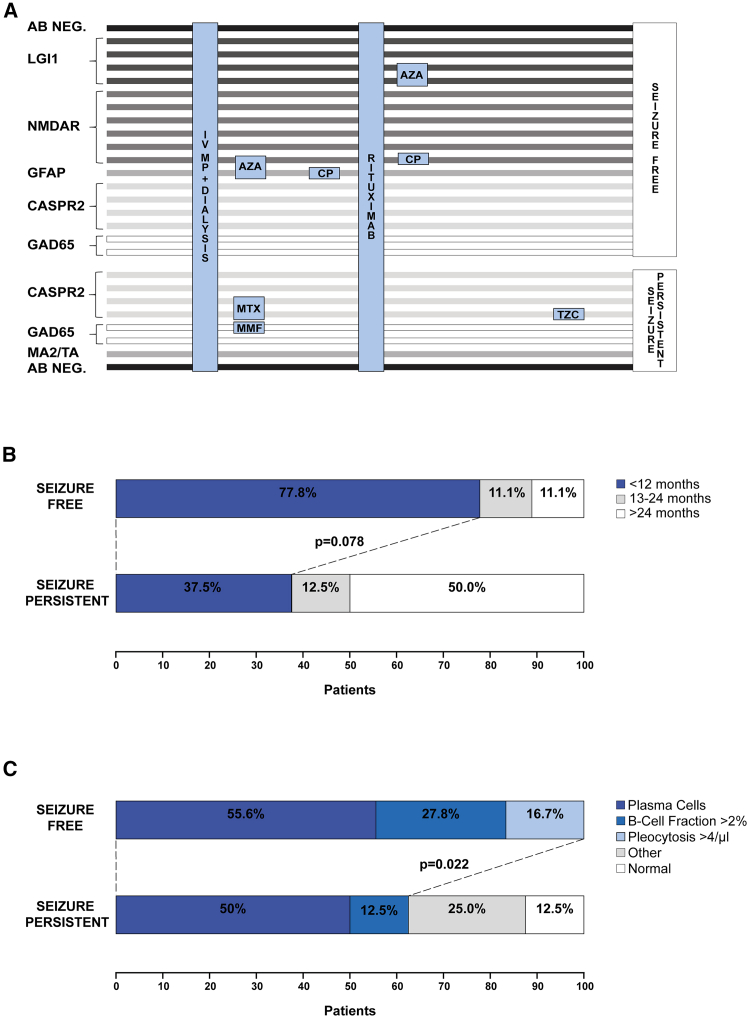
Timing and immune context of rituximab treatment (A) Sankey diagram showing immunosuppressive treatments received by the patients in the rituximab cohort, stratified by seizure outcome (seizure free: *n* = 18, seizure persistent: *n* = 8). (B) Distribution of time from disease onset to rituximab initiation in seizure-free and seizure-persistent patients. (C) Distribution of CSF findings in seizure-free and seizure-persistent patients. Antibody subtypes present within the respective categories are summarized in Table S1. CSF findings were categorized as plasma cells, B cell fraction >2%, pleocytosis >4/μL, other abnormalities, or normal CSF. “Other” included blood-brain barrier disturbance, elevated protein, or abnormal flow cytometry findings without B cell pathology. Categories were ordered according to CSF immune characteristics. *p* values were calculated using Fisher’s exact test. AZA, azathioprine; CP, cyclophosphamide; CSF, cerebrospinal fluid; MMF, mycophenolate mofetil; MTX, methotrexate; RTX, rituximab; TCZ, tocilizumab.

Detailed information on all patients’ clinical and paraclinical characteristics as well as the rate of seizure freedom is summarized in Table S1. Seizure-free patients and seizure-persistent patients in RTX-positive and RTX-negative groups did not differ with regard to their basic clinical and paraclinical characteristics. With regard to AB status, all anti-LGI1 and anti-NMDAR AB-positive patients were seizure free after treatment with RTX, whereas anti-CASPR2, anti-GAD65, and AB-negative ALE were evenly distributed between the two groups, i.e., seizure-free and seizure-persistent groups, despite similarities in overall immunosuppressive treatment (Figure 2A).

In addition to these commonly reported AB subtypes, other antibodies were identified in the cohort, as detailed in Table S1, which provides the exact number of patients per AB subgroup along with their corresponding clinical outcomes. Given the small numbers within individual AB subgroups and the biological differences between surface and intracellular antigen-associated disease, these observations are descriptive and should be interpreted cautiously. Time to treatment onset with RTX and positive CSF findings, showed numerical enrichment toward a seizure-free outcome. We next analyzed time to treatment with RTX and CSF findings as factors affecting seizure freedom.

### Treatment timing and seizure outcomes

RTX treatment was initiated within 12 months after disease onset in 77.8% of patients (*n* = 14) in the seizure-free group, whereas only 37.5% of patients (*n* = 3) in the seizure-persistent group started with RTX within 12 months after disease onset (*p* = 0.078; Figure 2B). Of note, when a 16-month cutoff was applied, two more patients (total *n* = 16) were in the seizure-free group, suggesting that even later-onset treatment might affect a seizure-free outcome (Figure 2B). Consistently, the median time from disease onset to RTX initiation was shorter in seizure-free patients than in those with persistent seizures (6.5 vs. 22.5 months; Mann-Whitney *p* = 0.0893).

### CSF immune context and seizure outcomes

**Figure 3:**
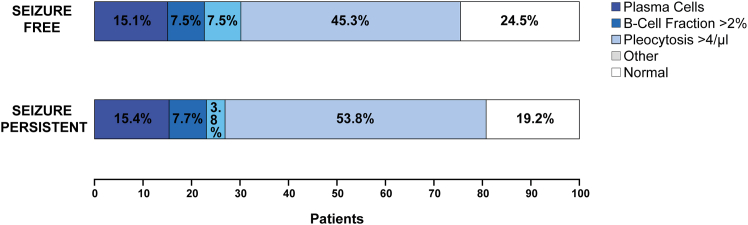
CSF immune profiles in patients without rituximab treatment The bar chart shows the distribution of CSF findings in seizure-free and seizure-persistent patients who did not receive rituximab treatment. Antibody subtypes present within the respective categories are summarized in Table S1. CSF findings were categorized as plasma cells, B cell fraction >2%, pleocytosis >4/μL, other abnormalities, or normal CSF. “Other” included blood-brain barrier disturbance, elevated protein, or abnormal flow cytometry findings without B cell pathology.

Seizure-free and seizure-persistent outcomes also differed depending on the CSF immune profile identified. Analyzing CSF, positive CSF findings (defined as plasma cells, elevated B cell fractions >2%, or pleocytosis >4/μL) were present in 100% of patients in the seizure-free group (*n* = 18), whereas this was only seen in 62.5% of patients in the seizure-persistent group (*n* = 5; *p* = 0.022; Figure 2C). Analyzing the categories in more detail, we found that overlap of categories, i.e., overlap of pleocytosis and plasma cells in CSF or elevated B cell fractions, respectively, was seen overall in ∼50% (9/20 patients). To exclude that seizure-free outcome is associated with specific CSF characteristics treatable with any other first-line immunosuppression like methylprednisolone, we also analyzed the distribution of positive CSF findings in patients who were not treated with RTX. Positive CSF findings were evenly distributed between seizure-free patients and patients who experienced ongoing seizures (Figure 3).

### Stratified analysis of rituximab treatment according to timing and immune context

**Figure 4:**
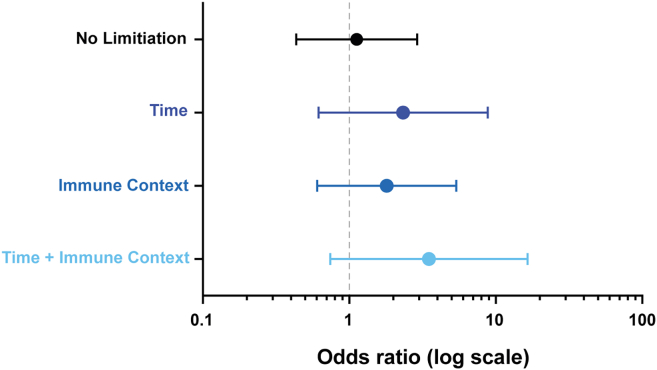
Odds ratios for seizure freedom after rituximab treatment (exploratory) The plot displays odds ratios ([ORs], dots) and 95% confidence intervals (horizontal lines) for seizure freedom after RTX treatment across four conditions: no preselection, early treatment timing, positive CSF immune context, and combined early treatment timing plus positive CSF immune context. The OR for each condition is shown on a logarithmic scale.

To gain further insight into the clinical relevance of our findings, we compared the efficacy of RTX treatment on seizure freedom, measured by odds ratios (OR) between the treated (RTX) and the control group (no RTX). First, we compared the patients who were treated with RTX according to seizure freedom without pre-selection (no limitations). The OR of 1.125 (95% confidence interval [CI], 0.434–2.916) indicates that there is a numerically slightly higher odds of seizure freedom with RTX, which was not statistically significant (Figure 4). When treatment onset within 12 months (Time) was considered, the OR of 2.333 (95% CI, 0.617–8.821) suggests that RTX was associated with numerically higher odds of achieving seizure freedom when treatment was initiated in a timely manner, in comparison to no RTX. A similar picture was observed when focusing on CSF-defined immune context for pre-selection. The OR of 1.8 (95% CI, 0.603–5.371) was associated with numerically higher odds of seizure freedom with RTX compared to no RTX. Upon combining the two selection factors, we observe an increase in the OR to 3.5 (95% CI, 0.741–16.523), indicating numerically higher odds of achieving seizure freedom, although CIs were wide and the estimates remained statistically non-significant. Overall, these exploratory analyses should be interpreted cautiously, as all estimates were imprecise, based on small subgroup sizes, and derived from a limited number of RTX-treated patients. However, they may be regarded as a valuable starting point for future studies designed to assess these findings in larger cohorts.

### Multivariable analysis of seizure freedom

To assess whether the observed associations persist after adjustment for potential confounders, we performed a multivariable logistic regression analysis in the entire cohort (*n* = 107), including demographic, clinical, imaging, and immunological variables. In the adjusted model, RTX treatment was not independently associated with seizure freedom (OR, 0.69; 95% CI, 0.14–3.36; *p* = 0.644).

Among clinical covariates, daily seizure frequency prior to immunotherapy (vs. no seizures) was strongly associated with seizure outcome (OR, 13.43; 95% CI, 1.69–106.80; *p* = 0.014), while older age at onset showed a modest but statistically significant association (OR, 1.04 per year; 95% CI, 1.01–1.08; *p* = 0.020).

Other variables, including AB group, MRI and EEG findings, affective and cognitive symptoms, blood-brain barrier disturbance, cytostatic therapy, and time to first-line immunotherapy initiation, did not show statistically significant independent associations with seizure freedom. A full overview of all variables included in the multivariable model, including coefficients, odds ratios, CIs, and *p* values, is provided in Table S2.

Formal interaction models including RTX treatment and RTX-specific variables (e.g., time to treatment or CSF-defined immune context) did not converge due to sparse subgroup sizes and separation of outcomes. However, stratified analyses (Figures 2 and 4) suggest that the association between RTX treatment and seizure freedom is context dependent, varying according to treatment timing and the underlying immunological characteristics.

## Discussion

The findings of this study provide insights into the potential associations between RTX treatment and seizure outcomes in ALE, using seizure freedom as a clinically meaningful outcome measure. This focus aligns with current conceptual distinctions between acute symptomatic seizures during active autoimmune encephalitis and autoimmune-associated epilepsy with enduring seizure predisposition.^1^^,^^2^ While global disability scales such as the modified Rankin scale remain widely used, they may insufficiently capture key non-motor manifestations in autoimmune encephalitis; composite disease-specific instruments such as the CASE score have been developed and validated and include seizure activity as a core domain.^6^^,^^7^ Although statistical significance was not achieved for OR analyses due to the limited number of RTX-treated patients, the subgroup findings should be interpreted with considerable caution, as the small RTX-treated cohort substantially limits the robustness and generalizability of these exploratory observations. Accordingly, these findings should not be interpreted as evidence of a definitive treatment effect, but rather as exploratory associations observed in a retrospective cohort. Consistent with this, RTX treatment was not independently associated with seizure freedom in the multivariable model, suggesting that any apparent benefit may depend on treatment timing and immunological context rather than reflecting a uniform treatment effect across a heterogeneous patient population.

We hypothesized that earlier initiation of RTX is associated with a higher likelihood of seizure freedom. Given that this cohort included patients with chronic or slowly progressive AB-associated ALE, such as anti-GAD65 and anti-CASPR2, a 12-month cutoff was chosen to define earlier treatment relative to disease biology. Patients who received RTX within this time frame showed a numerically higher likelihood of achieving seizure freedom compared to those treated later, suggesting that treatment timing may influence the outcome. Importantly, the term “early” in this study refers to treatment timing in relation to the often indolent disease course observed in this cohort and should not be equated with acute escalation strategies used in fulminant autoimmune encephalitis, where RTX is commonly considered early as a second-line therapy after failure of first-line immunotherapy.^3^^,^^4^^,^^5^ Notably, seizure freedom was also observed in individual patients treated beyond 12 months, up to 16 months after disease onset, although the apparent benefit diminished with increasing treatment delay.

Cellular CSF profiles prior to RTX treatment emerged as another relevant factor associated with seizure outcome. All patients who became seizure free after RTX exhibited positive CSF findings, whereas such profiles were present in only 62.5% of patients with persistent seizures. Mechanistically, RTX depletes CD20-expressing B cells, thereby reducing antigen presentation, cytokine production, and downstream T cell activation, which may contribute to attenuation of neuroinflammation and seizure generation. While plasma cells are not directly targeted, their presence in CSF may reflect ongoing B-cell-driven immune responses that are indirectly modulated by upstream B-cell depletion.^5^^,^^12^ However, these mechanistic considerations remain speculative and cannot be conclusively addressed in the present retrospective study. Similarly, pleocytosis alone does not constitute a specific RTX target but may serve as a surrogate marker indicating an inflammatory CNS milieu that may be associated with differential RTX responsiveness. Importantly, reliance on standard CSF analysis alone may miss RTX-relevant immune activity, whereas the combination of routine CSF parameters with CSF flow cytometry improves immunophenotypic resolution in autoimmune encephalitis and limbic encephalitis.^8^^,^^9^ These CSF-derived parameters should not be interpreted as direct therapeutic targets of RTX, but rather as indirect markers of an underlying immune activation state.

The role of AB profiles is also noteworthy. Patients with anti-LGI1 and anti-NMDAR antibodies uniformly achieved seizure freedom following RTX treatment, extending prior observations that have predominantly emphasized functional outcomes in these entities.^3^^,^^4^ At the same time, anti-LGI1 encephalitis often responds strongly to corticosteroids during the acute phase, highlighting that seizure outcomes may reflect AB- and stage-specific treatment responsiveness.^13^ In contrast, no clear association between AB status and seizure outcome was observed in patients with other AB-associated or AB-negative ALE, suggesting that disease severity, immunopathological mechanisms, or timing of treatment escalation may differ between these entities. These findings support the concept that selected AB-mediated disease processes may show differing associations with RTX treatment, while its role in other forms of ALE remains less clear. However, these observations are based on descriptive subgroup analyses and should be interpreted with caution. In the multivariable model, AB group was not independently associated with seizure freedom, suggesting that the observed differences between AB subtypes may be influenced by other clinical and treatment-related factors.

The inability to formally model interaction effects reflects the limited sample size and complete separation in key subgroups, further underscoring that subgroup-specific observations should be interpreted cautiously and considered exploratory.

A particular strength of this study is that the single-center design enabled detailed longitudinal characterization of seizure outcomes, EEG findings, MRI evolution, CSF immune profiles, and treatment trajectories across the disease course. Such comprehensive datasets are rarely available in large administrative databases and may be difficult to harmonize across multicenter cohorts, particularly in rare neuroimmunological disorders such as ALE.

### Limitations of the study

This study has several limitations. The inclusion of patients with heterogeneous AB profiles, including surface, intracellular, and AB-negative cases, represents an important source of clinical and biological heterogeneity, as these entities differ substantially in immunopathogenesis and treatment responsiveness. This heterogeneity limits the interpretability of subgroup-specific observations and may confound treatment-related associations. Its retrospective single-center design and the limited number of RTX-treated patients reduce statistical power and preclude causal inference. In particular, the small number of RTX-treated patients limited statistical power, resulted in wide confidence intervals, and restricted the robustness of subgroup-specific estimates. The imbalance between RTX-treated and non-RTX-treated patients reflects evolving treatment strategies over the long inclusion period and limits direct comparability between groups. The inclusion period (2005–2024) spans substantial changes in diagnostic criteria, AB testing methods, and treatment strategies in autoimmune encephalitis. These temporal developments may have influenced patient selection, thresholds for RTX initiation, recognition of AB-associated syndromes, and clinical outcomes. The definition of “early” RTX initiation (<12 months) was chosen relative to the disease biology of this cohort, which included chronic and slowly progressive AB-associated ALE, and, therefore, does not correspond to acute treatment windows applied in studies of fulminant autoimmune encephalitis.^3^^,^^5^ Seizure freedom was assessed clinically based on structured longitudinal follow-up and may be subject to recall bias, although complemented by serial EEG and long-term EEG recordings where available. Initial disease severity measures such as intensive care unit admission or nadir modified Rankin scale were not systematically analyzed, as the primary outcome of this study was seizure freedom rather than global functional outcome. Finally, the absence of an independent validation cohort limits generalizability and highlights the need for prospective multicenter studies to confirm these findings.

In addition, treatment allocation was not randomized, and the decision to initiate RTX may have been influenced by disease severity, response to first-line immunotherapy, symptom progression, and physician preference, introducing potential indication bias and limiting comparability between RTX-treated and non-treated patients.

Taken together, these real-world data suggest that consideration of both treatment timing and immunological context may be relevant when evaluating RTX treatment strategies in ALE with seizures and may help guide more individualized immunotherapeutic approaches.

## Resource availability

### Lead contact

Further information and requests for resources and data should be directed to and will be fulfilled by the lead contact, Stjepana Kovac (stjepana.kovac@ukmuenster.de).

### Materials availability

This study did not generate new unique reagents.

### Data and code availability

• Data: Data reported in this paper will be shared by the lead contact upon request. Due to patient privacy and ethical restrictions, the datasets are not publicly deposited and are available only subject to institutional and ethical approval.
• Code: This paper does not report original code.
• Other items: Any additional information required to reanalyze the data reported in this paper is available from the lead contact upon request.

## Acknowledgments

The authors thank all patients and clinical staff involved in this study. Funding: no specific funding was received for this work.

## Author contributions

A.D. and S.K. had full access to all of the data in the study and take responsibility for the integrity of the data and the accuracy of the data analysis. Concept and design, A.D., S.K., and S.G.M.; acquisition, analysis, or interpretation of data, A.D., S.K., N.G., A.K., R.D., M.P., and S.G.M; drafting of the manuscript, S.K., A.D., and L.B.; critical revision of the manuscript for important intellectual content, S.K., A.D., C.C.G., G.M.z.H., H.W., A.B., C.E., and S.G.M.; statistical analysis, A.D., L.B., N.L., and S.G.M.; obtained funding, none; administrative, technical, or material support, A.D., S.K., and T.B.; study supervision, A.D. and S.K.

## Declaration of interests

S.K. reported receiving grants from Deutsche Forschungsgemeinschaft and consultant honoraria from UCB, Jazz Pharmaceuticals, Eisai, and Angelini Pharma. G.M.z.H. was supported by a grant from the Bundesministerium für Bildung und Forschung (BMBF) “Lipid Immune Neuropathy Consortium” and by grants from the Deutsche Forschungsgemeinschaft (DFG) (ME4050/12–1, ME4050/13–1). S.G.M. receives honoraria for lecturing and travel expenses for attending meetings from Academy 2; Argenx; Alexion; Almirall; Amicus Therapeutics, Germany; Bayer Health Care; Biogen; BioNtech; BMS; Celgene; Datamed; Demecan; Desitin; Diamed; Diaplan; DIU Dresden; DPmed; Gen Medicine and Healthcare Products; Genzyme; Hexal AG; IGES; Impulze GmbH; Janssen Cilag; KW Medipoint; MedDay Pharmaceuticals; Medudy; Merck Serono; MICE; Mylan, Neuraxpharm, Neuropoint; Novartis; Novo Nordisk; ONO Pharma; Oxford PharmaGenesis; QuintilesIMS; Roche; Sanofi-Aventis; Springer Medizin Verlag; STADA; Chugai Pharma; Teva; UCB; Viatris; Wings for Life international; and Xcenda. His research is funded by the German Ministry for Education and Research (BMBF), Bundesinstitut für Risikobewertung (BfR), Deutsche Forschungsgemeinschaft (DFG), Else Kröner Fresenius Foundation, Gemeinsamer Bundesausschuss (G-BA), German Academic Exchange Service, Hertie Foundation, Interdisciplinary Center for Clinical Studies (IZKF) Muenster, German Foundation Neurology and Alexion, Almirall, Amicus Therapeutics Germany, Biogen, Diamed, DGM e.v., Fresenius Medical Care, Genzyme, Gesellschaft von Freunden und Förderern der Heinrich-Heine-Universität Düsseldorf e.V., HERZ Burgdorf, Merck Serono, Novartis, ONO Pharma, Roche, and Teva.

## Declaration of generative AI and AI-assisted technologies in the writing process

During the preparation of this work, the authors used ChatGPT (OpenAI) to support language editing and improve clarity. After using this tool, the authors reviewed and edited the content as needed and take full responsibility for the content of the published article.

## STAR★Methods

### Key resources table

**Table:** 

REAGENT or RESOURCE	SOURCE	IDENTIFIER
**Biological samples**		

Patient cohort (*n* = 107 ALE patients)	Department of Neurology, University Hospital Münster	This study

**Deposited data**		

Clinical dataset	This study	Available from the Lead Contact upon request; not publicly deposited due to ethical and data protection restrictions.

**Software and algorithms**		

IBM SPSS Statistics (v29.0.0.0)	IBM	https://www.ibm.com/products/spss-statistics
GraphPad Prism (v10.2.3)	Dotmatics	https://www.graphpad.com
MedCalc Odds Ratio Calculator (v23.0.9)	MedCalc Software Ltd	https://www.medcalc.org
Adobe Illustrator (v23.0.6)	Adobe	https://www.adobe.com
Scalable Vector Graphics (SVG 1.1)	W3C	https://www.w3.org/Graphics/SVG/

### Experimental model and study participant details

We retrospectively enrolled 107 consecutive immunotherapy-naïve patients treated at the Department of Neurology, University of Münster. The cohort comprised adult female and male patients. Sex was recorded as part of the clinical data collection and is reported descriptively in Table S1. Patient selection, inclusion and exclusion criteria, as well as data availability and processing are described in the main manuscript (Figure 1). Patients were included based on clinical features consistent with autoimmune limbic encephalitis, aligned with established diagnostic frameworks for autoimmune encephalitis, including Graus et al. (2016), and supported by clinical, MRI, EEG, and CSF findings. In clinical practice, rituximab treatment was generally initiated in patients with ongoing disease activity, insufficient response to first-line immunotherapy, recurrent or persistent seizures, or a severe clinical course supported by CSF, MRI, and EEG findings. The decision to administer rituximab was based on an individualized clinical assessment and may have been influenced by disease severity, response to first-line immunotherapy, symptom progression, and physician judgment. Patients were not prospectively allocated to treatment groups; RTX treatment reflected routine clinical decision-making.

In brief, 156 patients were initially screened, of whom 49 were excluded due to insufficient follow-up (< 12 months), competing diagnoses, seizures of non-limbic origin, lack of immunotherapy, or pre-existing hippocampal sclerosis.

The final cohort comprised 107 patients fulfilling all inclusion criteria.

All patients underwent comprehensive clinical work-ups which included longitudinal EEG and long-term EEG measurements, serial MRIs and CSF analyses in the course of the disease including detailed flow cytometry analyses in blood and CSF as described in detail in our previous work.^14^^,^^15^

The extent of available data per patient, including clinical visits, imaging, electrophysiology, and CSF analyses, is summarized in the main manuscript (Figure 1).

The study was approved by the ethics committee of the University of Münster (registration nos. 2010-262-f-S, 2013-682-b-S, 2013-350-f-S, 2016-053-f-S, 2017-754-f-S, 2019-712-f-S). All patients signed written informed consent.

### Method details

Time to treatment start with Rituximab was stratified into four categories (0–6, 7–12, 13–24 and > 24 months).

The definition of “early” RTX initiation (< 12 months) was chosen relative to the disease biology of this cohort, which included patients with chronic or slowly progressive antibody-associated ALE (e.g., anti-GAD65 and anti-CASPR2), and therefore does not correspond to the acute treatment windows applied in studies of fulminant autoimmune encephalitis.

We classified CSF as “positive” if it contained cellular features interpreted as surrogate markers of immune activation potentially associated with responsiveness to peripheral B-cell depletion. Specifically, criteria included:

1. Detection of plasma cells,
2. Elevated B-cell fractions ( >2%), or
3. Pleocytosis (> 4/μL).

Pleocytosis alone was not considered a direct RTX target, but rather a surrogate marker of intrathecal inflammation potentially associated with B-cell-driven immune activity.

Plasma cells detected in CSF do not represent CD20-expressing cells, but were interpreted as markers of an ongoing B-cell–driven immune response upstream of CD20-positive B cells. Importantly, these CSF-derived parameters were not interpreted as direct therapeutic targets of rituximab, but rather as indirect markers of an underlying immune activation state potentially associated with treatment responsiveness.

Seizure freedom was defined clinically as absence of seizures for more than 12 months from the date of the last visit, based on structured follow-up; EEG findings were reviewed where available.

Data processing included stratification according to seizure outcome and rituximab treatment, as well as analysis of more than 20 clinical and paraclinical variables (Figure 1).

Antibodies were grouped based on their presumed pathogenic mechanisms into surface antibodies (LGI1, CASPR2, NMDAR, neuropil, and VGKC) and intracellular antibodies (Hu, Yo, Ma2/Ta, Zic4, Drebrin, GFAP, and GAD65). VGKC antibodies were not considered a distinct pathogenic entity. VGKC positivity was interpreted cautiously and in the context of possible LGI1 or CASPR2 reactivity.

### Quantification and statistical analysis

Statistical analysis was performed using IBM SPSS Statistics (version 29.0.0.0).

Statistical analyses were performed to identify independent predictors of seizure freedom using multivariable logistic regression.

The primary outcome variable was seizure freedom for ≥12 months (binary outcome). The main exposure of interest was rituximab treatment (yes/no).

A multivariable logistic regression model was fitted in the entire cohort, including the following covariates selected *a priori* based on clinical relevance: age at disease onset, seizure frequency prior to immunotherapy initiation, affective symptoms prior to immunotherapy, cognitive symptoms prior to immunotherapy, antibody group (categorized as described above), MRI abnormalities of the limbic system (collapsed into normal vs. abnormal, including enlargement, hyperintensity, or sclerosis), bilateral MRI involvement, EEG abnormalities (collapsed into normal vs. abnormal, including epileptic discharges or slowing), bitemporal EEG focus, blood–brain barrier disturbance, use of cytostatic therapy, time from disease onset to initiation of first-line immunotherapy (months).

All variables were entered simultaneously into the multivariable model.

To explore whether the effect of rituximab depends on clinical context, additional exploratory analyses were conducted within the rituximab-treated subgroup. These included:

• Comparison of seizure outcomes according to the presence of CSF-derived immune features
• Analysis of time from disease onset to rituximab initiation (continuous and dichotomized at 12 months)

Formal interaction terms (e.g., rituximab × time to treatment, rituximab × immune context) were tested but not included in the final model due to lack of model convergence, caused by sparse subgroup sizes and complete separation of outcomes.

Categorical variables were compared using Fisher’s exact test, and continuous variables using the Mann–Whitney U test.

Odds ratios (ORs) with 95% confidence intervals (CIs) were reported.

Reported odds ratios represent adjusted estimates from the multivariable model.

Odds ratios were additionally computed using MedCalc Software Ltd. (https://www.medcalc.org/calc/odds_ratio.php; Version 23.0.9; accessed November 29, 2024).

A two-sided *p* value <0.05 was considered statistically significant; all analyses were considered exploratory due to the retrospective design and limited sample size.

The Sankey diagram was constructed with Scalable Vector Graphics (version 1.1 s edition).

Bar graphs were generated using IBM SPSS Statistics (version 29.0.0.0).

The odds ratio diagram was created with GraphPad Prism (version 10.2.3).

All figures were processed with Adobe Illustrator 2019 (version 23.0.6).
