# Supplementary material for: Immune context and treatment timing associated with seizure freedom after rituximab in autoimmune limbic encephalitis
Source: iScience. 2026 Jul 10;29(8):116689. doi: 10.1016/j.isci.2026.116689 (PMC13380735; doi:10.1016/j.isci.2026.116689)
Supplement: Document S1. Tables S1 and S2 [file mmc1.pdf]

iScience, Volume *29*

Supplemental information

Immune context and treatment timing associated with seizure freedom after rituximab in autoimmune limbic encephalitis

Andre Dik, Nils Landmeyer, Noëmi Gmahl, Laura Bierhansl, Rakshit Dadarwal, Alina Kosfeld, Matthias Pawlowski, Tobias Brix, Catharina C. Gross, Gerd Meyer zu Hörste, Heinz Wiendl, Antje Bischof, Christian Elger, Sven G. Meuth, and Stjepana Kovac

Supplemental Table S1. Demographics, clinical features, diagnostic findings, and therapy in patients treated with rituximab compared with patients without rituximab, stratified by seizure outcome.

Variables	Rituximab Group n = 26			No Rituximab Group n = 81		
	Seizure free (n=18)	Seizure persistent (n=8)	p-value	Seizure free (n=54)	Seizure persistent (n=27)	p-value
**Demographics**						
Gender, female n (%)	10 (55.6)	3 (37.5)	.673	22 (40.7)	10 (37)	.813
Age at onset, median, years (IQR)	44 (36.5)	43 (34.5)	.723	57.5 (19.5)	42 (31)	.008
Follow-up time, median, months (IQR)	35 (24)	62.5 (70)	.118	41 (45.25)	38 (34)	.790
**Clinical features, before IT-start**						
Seizure frequency			0.76			.003
daily, n (%)	8 (44.4)	2 (25)	n.s.	20 (37)	2 (7.4)	.005
weekly, n (%)	3 (16.7)	1 (12.5)	n.s.	5 (9.3)	9 (33.3)	n.s
monthly, n (%)	1 (5.6)	1 (12.5)	n.s.	9 (16.7)	3 (11.1)	n.s
<1x/month, n (%)	6 (33.3)	4 (50)	n.s.	20 (37)	13 (48.1)	n.s
Status epilepticus, n (%)	5 (27.8)	1 (12.5)	.628	6 (11.1)	2 (7.4)	.712
Affective disorders, n (%)	14 (77.8)	4 (50)	.197	33 (61.1)	15 (55.6)	.640
Cognitive disorders, n (%)	17 (94.4)	6 (75)	.215	41 (75.9)	19 (70.4)	.601
Autonomic disorders, n (%)	9 (50)	5 (62.5)	.683	14 (25.9)	9 (33.3)	.602
Viral prodromal symptoms, n (%)	4 (22.2)	2 (25)	1.00	3 (5.6)	1 (3.7)	1.00
Malignancy, n (%)	3 (16.7)	0 (0)	.529	12 (22.2)	5 (18.5)	.779
Autoimmune disorder, n (%)	3 (16.7)	3 (37.5)	.330	15 (27.8)	6 (22.2)	.789
Polyneuropathy, n (%)	4 (22.2)	3 (37.5)	.635	14 (25.9)	10 (37)	.315
**Diagnostic values, before IT-start**						
Antibody present, n (total)	17 (18)	7 (8)	.529	16 (54)	10 (27)	.615
NMDAR, n	6	0		0	0	
CASPR2, n	4	4		1	1	
LGI1, n	4	0		7	1	
GAD65, n	2	2		1	5	
other, name	GFAP	Ma2/Ta		Hu, 2xNeuropil, VGKC, Yo, 2xZic4	Drebrin, Hu, Neuropil	
Graus classification						
definite	3	1		8	4	
probable	2	1		6	5	
possible	1	2		4	3	
MRI of the limbic system			.628			.027
normal, n (%)	5 (27.8)	1 (12.5)		7 (13)	4 (14.8)	n.s.
sclerosis, n (%)	0 (0)	0 (0)		1 (1.9)	5 (18.5)	.007
enlargement/hyperintensity, n (%)	13 (72.2)	7 (87.5)		46 (85.2)	18 (66.7)	n.s.
bilaterally affected, n (%)	9 (69.2)	4 (57.1)		16 (34.8)	8 (44.4)	n.s.

Continued

Supplemental Table S1. Continued

Variables	Rituximab Group n = 26			No Rituximab Group n = 81		
	Seizure free	Seizure persistent	p-value	Seizure free	Seizure persistent	p-value
EEG			.525			.592
normal, n (%)	3 (16.7)	1 (12.5)		5 (9.3)	1 (3.7)	
slowing, n (%)	8 (44.4)	2 (25)		6 (11.1)	4 (14.8)	
epileptic discharges, n (%)	7 (38.9)	5 (62.5)		43 (79.6)	22 (81.5)	
bitemporal focus, n (%)	8 (44.4)	4 (50)		22 (51.2)	6 (27.3)	
CSF						
Pleocytosis >4/µl	9 (50)	3 (37.5)	.683	9 (17)	1 (3.8)	.153
BBB disturbance, n (%)	8 (44.4)	2 (25)	.420	16 (30.2)	11 (42.3)	.320
OCB			.798			.095
Type 1, n (%)	11 (61.1)	5 (62.5)		40 (75.5)	23 (88.5)	
Type 2, n (%)	6 (33.3)	2 (25)		8 (15.1)	0 (0)	
Type 4, n (%)	1 (5.6)	1 (12.5)		3 (6.4)	1 (4)	
**Therapy**						
ASM overall			.197			.096
1-2, n (%)	14 (77.8)	4 (50)		33 (62.3)	11 (40.7)	
>2, n (%)	4 (22.2)	4 (50)		20 (37.7)	16 (59.3)	
Methylprednisolone			.828			.915
1-15 g, n (%)	15 (83.3)	6 (75)		30 (55.6)	14 (51.9)	
16-30 g, n (%)	2 (11.1)	1 (12.5)		18 (33.3)	9 (33.3)	
>30 g, n (%)	1 (5.6)	1 (12.5)		4 (7.4)	2 (7.4)	
Dialysis, n (%)			.103			.712
1-5, n (%)	7 (38.9)	1 (12.5)		9 (16.7)	4 (14.8)	
6-15, n (%)	11 (61.1)	5 (62.5)		5 (9.3)	5 (18.5)	
>15, n (%)	0 (0)	1 (12.5)		4 (7.4)	2 (7.4)	
Cytostatics						
MTX, n	0	2		3	5	
CP, n	2	0		1	1	
AZA, n	4	0		7	2	
other, name	0	1xMMF, 1xTCZ		1xCTX	1xMMF	
Time from onset to first IT			.321			.206
0-6 months, n (%)	16 (88.9)	5 (62.5)		17 (31.5)	6 (22.2)	
7-12 months, n (%)	1 (5.6)	1 (12.5)		11 (20.4)	2 (7.4)	
13-24 months, n (%)	1 (5.6)	1 (12.5)		5 (9.3)	5 (18.5)	
>24 months, n (%)	0 (0)	1 (12.5)		21 (38.9)	14 (51.9)	
Rituximab cycles, median (IQR)	4 (3.25)	3 (4.75)	.905	0	0	

Note: Data are presented as median (IQR) or n (%). ASM = anti-seizure medication; AZA = azathioprine; BBB = blood–brain barrier; CP = cyclophosphamide; CTX = chemotherapy; IT = immunotherapy; MMF = mycophenolate mofetil; MTX = methotrexate; OCB = oligoclonal bands; RTX = rituximab; TCZ = tocilizumab; n.s. = not significant. P values are two-tailed and Bonferroni corrected for multiple comparisons. Group sizes are indicated in the column headers.

Comparisons between the rituximab (RTX) and no-rituximab groups were performed separately within the seizure-free and seizure-persistent subgroups. Antibody-negative patients were classified according to the Graus et al. criteria (2016) into definite, probable, and possible autoimmune encephalitis based on clinical presentation, MRI, EEG, and CSF findings. This classification was used to further characterize the heterogeneity of antibody-negative cases

within the cohort. Categorical variables were compared using the chi-square test or Fisher's exact test, as appropriate; continuous variables were compared using the Mann-Whitney U test. P values are two-tailed and Bonferroni corrected.

Supplemental Table S2 Multivariable logistic regression analysis of factors associated with seizure freedom				
**Variable**	**Comparison**	**OR**	**95% CI**	**p-value**
Rituximab treatment	yes vs no	0.69	0.14–3.36	0.644
Age at onset (years)	per year	1.04	1.01–1.08	0.020
Seizure frequency				
	<1x/month vs none	0.51	0.09–2.76	n.s.
	monthly vs none	3.12	0.29–33.80	n.s.
	weekly vs none	0.48	0.07–3.35	n.s.
	daily vs none	13.43	1.69–106.80	0.014
Antibody group				
	intracellular vs none/unknown	0.26	0.07–1.20	0.086
	surface vs none/unknown	1.13	0.28–5.52	0.880
	intracellular vs surface	0.23	0.04–1.27	0.092
MRI limbic abnormality	abnormal vs normal	1.73	0.28–10.73	n.s.
EEG abnormality	abnormal vs normal	0.15	0.02–1.44	0.057
Affective disorders	yes vs no	1.31	0.38–4.47	n.s.
Cognitive disorders	yes vs no	1.84	0.51–6.71	n.s.
MRI bilateral involvement	yes vs no	0.34	0.08–1.41	0.076
EEG bitemporal focus	yes vs no	2.04	0.54–7.68	n.s.
BBB disturbance	yes vs no	0.75	0.22–2.53	n.s.
Cytostatic therapy	yes vs no	0.40	0.12–1.37	n.s.
Time to first-line IT (months)	per month	0.99	0.97–1.00	0.151

Note: BBB = blood–brain barrier; CI = confidence interval; IT = immunotherapy; OR = odds ratio; n.s. = not significant. MRI abnormalities include enlargement, hyperintensity, or sclerosis; EEG abnormalities include slowing or epileptic discharges. Reference categories were: no rituximab treatment, no seizures prior to immunotherapy, antibody-negative/unknown, normal MRI, normal EEG, absence of clinical features, and no cytostatic therapy. Cytostatic therapy was defined as treatment with conventional immunosuppressive/cytotoxic agents (azathioprine, cyclophosphamide, mycophenolate mofetil, methotrexate, and tocilizumab), excluding rituximab. P-values are derived from Wald tests in the multivariable logistic regression model. Model fit was assessed using standard logistic regression diagnostics. No major violations of model assumptions or relevant multicollinearity among included covariates were identified. The model showed acceptable overall fit and calibration, although results should be interpreted cautiously given the limited sample size and number of events.

Supplemental Table S2 shows the results of the multivariable logistic regression analysis of factors associated with seizure freedom.

Odds ratios (ORs) with 95% confidence intervals (CIs) are presented for all variables included in the model and represent adjusted estimates.

The model included rituximab treatment, age at onset, seizure frequency prior to immunotherapy, affective and cognitive symptoms, antibody group, MRI and EEG findings, blood–brain barrier disturbance, cytostatic therapy, and time from disease onset to initiation of first-line immunotherapy.

Antibodies were categorized into surface antibodies (LGI1, CASPR2, NMDAR, neuropil, and VGKC-associated cases interpreted cautiously in the context of possible LGI1 or CASPR2 reactivity) and intracellular antibodies (Hu, Yo, Ma2/Ta, Zic4, Drebrin, GFAP, and GAD65), with antibody-negative patients serving as the reference group.

All analyses are exploratory and should be interpreted with caution given the retrospective design and limited sample size. Given the limited number of events, the model may be underpowered and results should be interpreted cautiously.
